# Influence of Surfactant for Stabilization and Pipeline Transportation of Iron Ore Water Slurry: A Review

**DOI:** 10.1021/acsomega.2c02534

**Published:** 2022-08-12

**Authors:** Mandakini Behari, Debadutta Das, Ardhendu Mouli Mohanty

**Affiliations:** † Department of Mechanical Engineering, Centurion University of Technology and Management, Bhubaneswar, Odisha 752050, India; ‡ Department of Chemistry, Basic Science & Humanities, Radhakrishna Institute of Technology and Engineering, IDCO-01, IDCO Industrial Estate, Barunei, Khordha, Odisha 752057, India

## Abstract

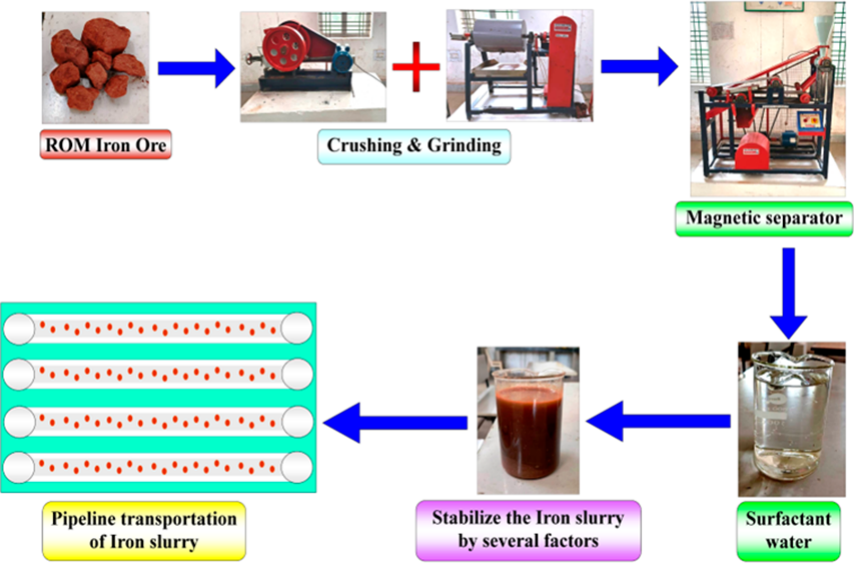

Iron ore is generally transported using a traditional method that releases significant amounts of dust into the environment. In contrast, the pipeline transportation of slurry is noticeably a sustainable approach for efficiently transporting iron ore by reducing the environmental pollution. The interparticle interaction of the iron ore particles should be mutually repulsive for steady dispersion. Surfactants and polymers adsorb efficiently at the solid/liquid interface due to their amphiphilic character, rendering the surface hydrophilic or hydrophobic to create a stable dispersion. The present review discusses the interaction of surfactants on the stabilization of solid particles for the ease of pipeline transportation using various types of stabilization mechanisms. In addition to the effect of surfactant alone, its combination with some other parameters such as particle size distribution, temperature, solid concentration, etc. has been discussed. The review also describes the detailed classification of iron ore, surfactant, and characteristic properties of surfactants.

## 1 Introduction

Slurry pipeline systems are widely utilized around the world as a feasible alternative to large-scale solids transport for conveying minerals such as fly ash,^1^ iron ore (IO), coal,^2,3^ lime stone,^4^ copper concentrate, zinc tailings,^5,6^ and other materials. IO fines play an important role in sintering^7,8^ and palletization.^9,10^ Currently, a large amount of IO fines have been transported from mining sites to the plant through the pipeline,^11^ which is both economically and environmentally beneficial. The slurry pipelines will go a long way toward decreasing pollution and traffic congestion. It is necessary to prepare a well-dispersed uniform suspension of IO particles in water as the transport medium before conveying the bulk slurry through pipelines. To negotiate the pumping power with the lowest specific energy, the rheological behavior of the concentrated slurry demands careful analysis concerning viscosity and other slurry flow parameters.^9,12−15^

The rheology of the slurry has been identified as an important criterion for determining the pressure drop requirements. As a result, studying the rheology of the slurry to predict the pressure drop and thus pumping efficiency would be beneficial.^16−18^ Surfactants have a significantly important role in the slurry transportation system. In addition, other parameters such as the temperature, solid concentration, slurry viscosity, and particle size distribution (PSD)^19−21^ affect the flow behavior of the iron water slurry (IWS).^22−24^ The interaction of dispersant or surface-active agents with the slurry particle impacts the flow behavior of the slurry. Therefore, surfactant selection is critically important. This study reviews and reports on the process by which the dispersant interacts with the slurry to reduce viscosity, the behavior of the slurry during transport, and the stabilization of various IWS systems.^11,25^ Although commercial surfactants are frequently used in slurry stabilization, greener approaches, such as the use of natural surfactants, seem to be promising and offer several benefits.^11^ The temperature affects fluid viscosity in addition to surfactant and PSD. The impact of temperature change on the apparent viscosity of CWS was examined using saponin as a dispersant that was extracted from *Sapindus laurifolia*.^26^ An increase in the kinetic energy of solid particles and rapid movement of the connected hydrophilic sugar unit chain of saponin may be the primary causes of viscosity reduction of the slurry.^20^

Different types of slurries, such as coal–water slurry, fly ash water slurry, oil–water emulsion, clay water slurry, and food slurry, have drawn interest in industrial applications.^27−29^ Coal–water slurry is a potential replacement for oil in several industrial applications and as an alternative fuel for the power sector.^27^ Because coal is a heterogeneous mixture of carbonaceous and mineral materials, its surface is largely hydrophobic and easily coalesces to form clusters, lowering the stability of the coal–water dispersion.^24,30^ Interparticle interaction of coal must be mutually repulsive for steady dispersion. Surfactants and polymers adsorb effectively on the solid/liquid interface because of their amphiphilic character, rendering the surface hydrophilic or hydrophobic. Based on the charge of the headgroups and the length of both the hydrophilic part and the hydrophobic chain, they perform as an electrostatic/steric hindrance for particle–particle contact. When adsorbed to the coal–water interface, numerous widely accessible surfactants and polymers increased the concentration of coal in the slurry and improved the stability of the coal–water slurry.^27^

Similar to the coal–water slurry, industrial application of fly ash slurry at high solid concentrations necessitates a thorough analysis of the rheological properties.^31^ A surfactant is added to the suspension to keep the solid particles in the dispersed phase during aqueous pipeline transportation, thereby reducing surface tension and increasing spreading and wetting qualities.^28^ Various kinds of surfactant and polymers such as sodium hexametaphosphate,^32^ cetyltrimethyl ammonium bromide (CTAB),^33^*Sapindus laurifolia*, and sodium dodecyl sulfate (SDS)^34^ have been developed by different researchers for stabilization of high concentration fly ash slurry. The key characteristic of the oil–water emulsion is of attainment of a stable state for its efficient adoption in the pipeline transport process, which can be validated based on achieving the ideal rheological properties, including viscosity, dispersion, and wettability behavior. In this emulsion stabilization system, the role of surfactants (dispersants) is crucial for attaining the above salient features. The addition of surfactants not only helps to decrease the interfacial tension between oil–water but also imparts the stabilization of the emulsion.^35,36^ In general, the dispersant possesses two moieties in its molecular skeleton: one is polar, and the other one is nonpolar, acting as a suitable one for improving the interfacial characteristics of the oil through adsorption of dispersants. When several advanced processes are applied for controlling the physical properties of the surrounding medium with the supplement of change in electrolytes,^37^ the pH of the emulsion and dispersant concentration appear to be causative on the effective stabilization of the oil–water emulsion to greater extent.^38^

Clay mineral–surfactant interactions are critical in many commercial applications, including water treatment, paints, and mineral flotation.^39,40^ Since clay minerals are often negatively charged, many researchers have focused on the interactions between cationic surfactants and clay.^41^ Different types of food surfactants are found to effectively stabilize food slurry. Surfactants interact with all of the primary ingredients of flour, such as starch, gluten, and lipids, and have a profound impact on their colloidal stability and emulsification process. Lecithin’s adaptability as an additive or emulsifier in food is a result of its nontoxicity. Lecithin facilitates the homogeneous mixing process, minimizes viscosity, substitutes more expensive chemicals, controls sugar solidification, and has coating potential.^42^ It prevents dust and aids in complete dispersion in water to adhere to surfaces. It also enhances the wetting qualities of lipophilic powders such as cocoa powder and hydrophilic powders such as low-fat proteins.

**Figure 1:**
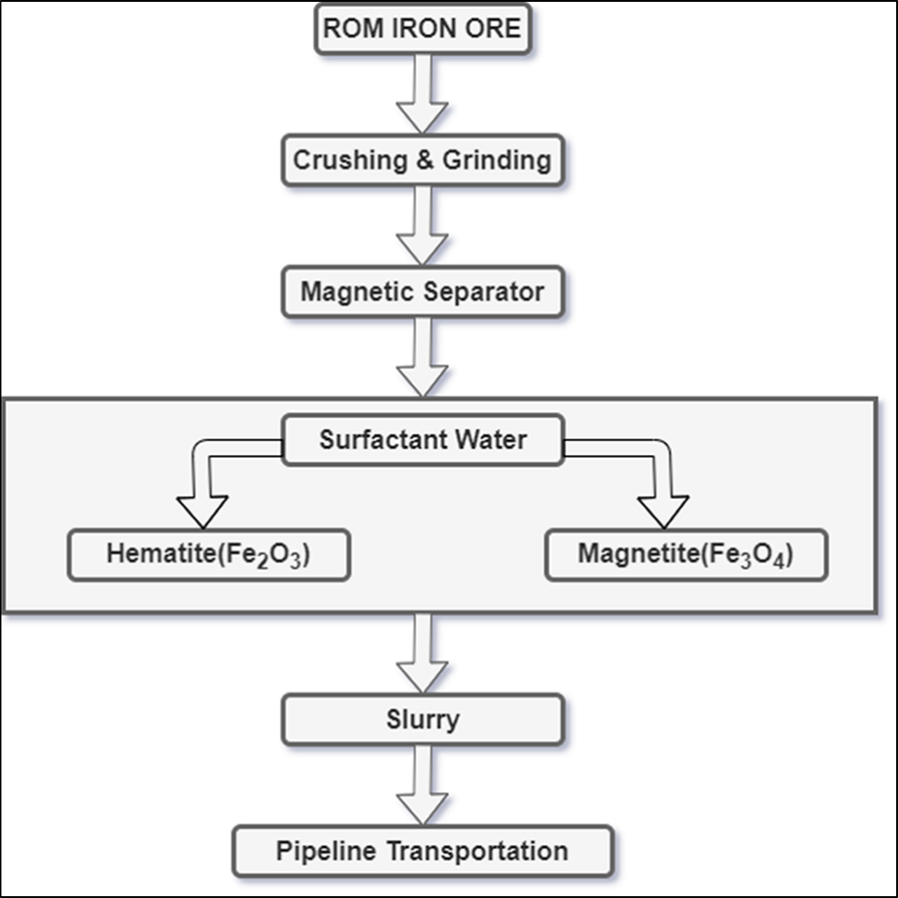
Schematic experimental setup for transportation of IWS.

A thorough review of the literature reveals that no review work on the systematic application of surfactant in the stabilization and transportation of IWS has been published. The present review is an attempt to cover the use of different surface-active agents in the stabilization of IWS. In addition to this, a systematic classification and characteristics of IO and surfactant are given. A schematic setup for transportation of IWS is represented in Figure 1.

## 2 About Iron Ore

In a variety of geologic contexts, IO can be found in igneous, metamorphic, or sedimentary rocks.^43,44^ Although the majority of IO are sedimentary, many have undergone weathering. This creates a problem to identify their exact origin. Oxides are the most prevalent iron-bearing minerals such as hematite (Fe_2_O_3_), which accounts for the vast majority of IO, which is red; magnetite (Fe_3_O_4_), being black; limonite or bog-IO (2Fe_2_O_3_·3H_2_O), is brown; and siderite (FeCO_3_), which is light brown (Table 1).^45,46^ The two most typical forms of ore are hematite and magnetite. Hematite sometimes goes by the label “natural ore”. The phrase refers to the early stages of mining since some hematite ores contained as much as 66% iron and could be fed into blast furnaces directly. Pure magnetite contains 72.4% iron, 59.8% limonite, and 48.2% siderite. The other components of ore (Table 2), referred to as gangue as a whole, can also affect the quality of the ore.^47^ White gangue minerals found in IO include quartz, feldspar, and calcite. These whitish gangue minerals have magnetic susceptibilities that are almost negligible. As a result, magnetic separation makes it very simple to separate them from IO. The majority of low-grade IO that is collected from the soil contains both useful and undesirable elements. Magnetic separation is the process of separating different minerals based on their variations in magnetic susceptibility by applying a magnetic field with the proper intensity, gradient, and other conditions. For efficient transportation, it is necessary to remove the gangue from the ore before transporting the IO.^48^

**Table 1:** Classification of IO Is Based on Physical Appearance

Sl no.	appearance	ore type
1	brown	hematite (Fe_2_O_3_)
2	black	magnetite (Fe_3_O_4_)
3	brown	limonite or Bog-IO (2Fe_2_O_3_·3H_2_O)
4	pale brown	siderite (FeCO_3_)

**Table 2:** Classification of IO According to the Quality of Associated Gangue

ore’s composition	associated gangue characteristics
siliceous	predominantly silica
aluminous	mainly alumina
argillaceous	clayey stuff is the most common kind
calcareous	lime is the most common kind of lime
bituminous	bituminous or coaly materials in large quantities
titaniferous	ilmenite in large quantities

### 2.1 World’s Top Five Largest IO-Producing Countries

**Figure 2:**
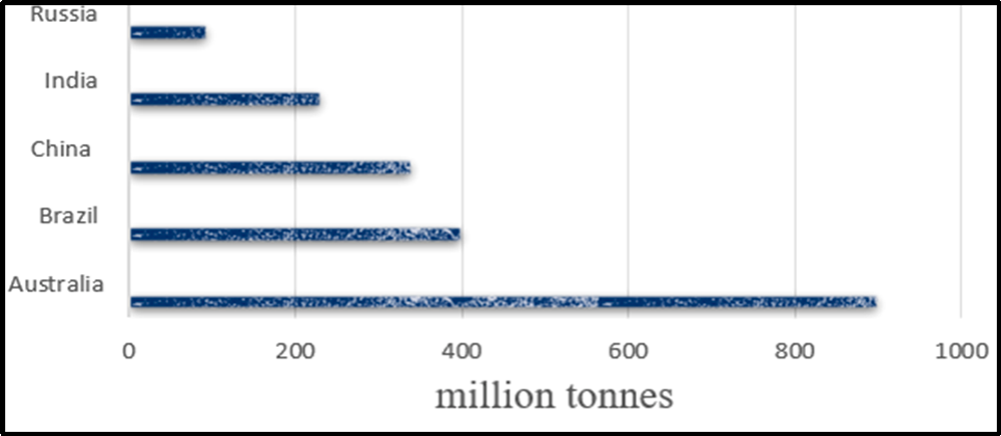
World’s top five largest IO-producing countries.

The world’s top five IO mining countries, namely, Australia, Brazil, China, India, and Russia accounted for more than 80% of global production in 2020 (Figure 2).^49^

## 3 Measurement of Viscosity of Iron Water Slurry

**Figure 3:**
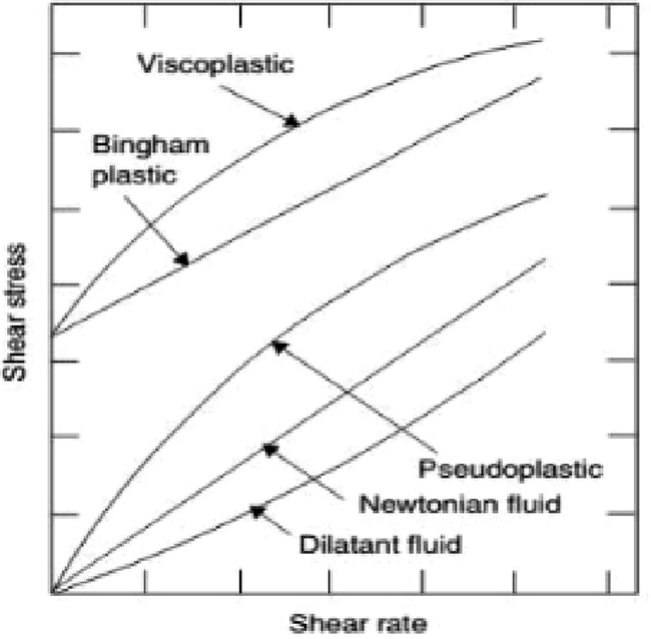
Classification of different types of fluids.

For a smooth slurry flow, the viscosity value should be reduced while being transported via a pipeline.^27,50^ When iron is added to water, the viscosity of the slurry inevitably increases compared to that with water alone. The laminar flow characteristic that directly links the velocity gradient to shear stress is suspension viscosity.^51^ Fluids are categorized as Newtonian or non-Newtonian depending on how they respond to shear stress and shearing rate. Taking shear stress and shear rate into account, Figure 3 depicts different kinds of fluid.^52^

## 4 Stability of Slurry: DLVO Theory of Colloidal Stability

The enhanced cohesive force among iron particles in the concentrated slurry increases the slurry’s viscosity. At higher concentrations, the iron particles flocculate and settle, causing the slurry to destabilize. With increased solid loading, the viscosity of the slurry increases, making pipeline transportation more difficult.^53,54^ As a result, for the concentrated slurry to be economically viable, a trade-off between optimal viscosity and stability is required. Because of the intense particle–particle association (hydrophobic–hydrophobic) that happens during the Brownian motion of iron particles, aggregation of particles occurs, resulting in flocculation and settling of iron particles.^55,56^ Thus, by hiding the hydrophobic site of iron or altering the iron surface, iron–water contact may be enhanced for a stable dispersion.^25,57^ A theory known as the DLVO theory is used to calculate mutual repulsion. It is described here since it is fundamental to slurry stabilization. Derjaguin, Landau, Verwey, and Overbeek formulated the DLVO hypothesis.^58−60^ According to this theory, a particle’s total potential energy VT, which is the sum of attracting contributions (VA), repulsive contributions (VR), and solvent potential energy (VS), determines the particle’s slurry stability. Although VS contributes the least to total potential energy. The stability of colloidal systems is primarily determined by VA and VR. Two types of forces emerge among particles, i.e., the van der Waals attractive force VA and the electrical double-layer repulsive forces VR.

**Figure 4:**
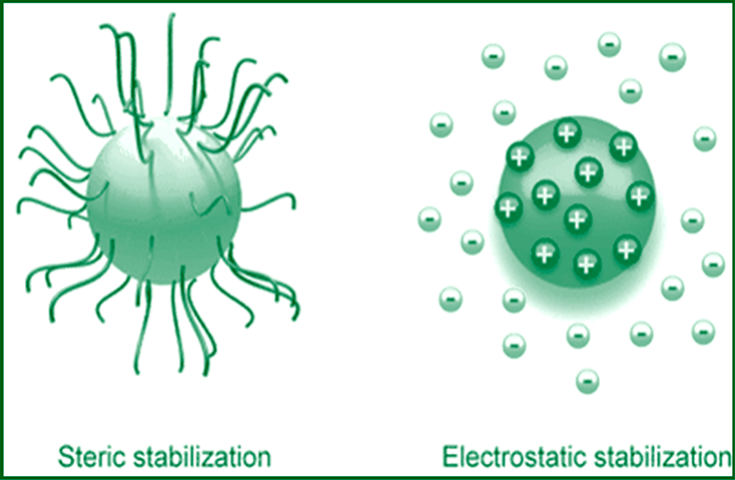
Two fundamental stabilizations.

- The two fundamental mechanisms that impact the stability of any dispersion are shown in Figure 4. Steric repulsion: When a dispersing agent adsorbs on a particle, the thickness of the dispersing agent’s coating at the particle’s surface causes steric repulsions, which reduce van der Waals forces of contact between solid particles. In this way, the particle does not come into contact with each other, and the adhesion between the particles is checked, as also the flocculation of the particles.^30^
- Electrostatic stabilization is defined as a distribution of charged species in a system that causes particle repulsion and hence system stabilization. Furthermore, the particle must be adequately wetted by the solvent to avoid particle–particle contact and therefore stabilize the dispersion.

## 5 Introduction to Surfactant, Classification, and Properties

**Figure 5:**
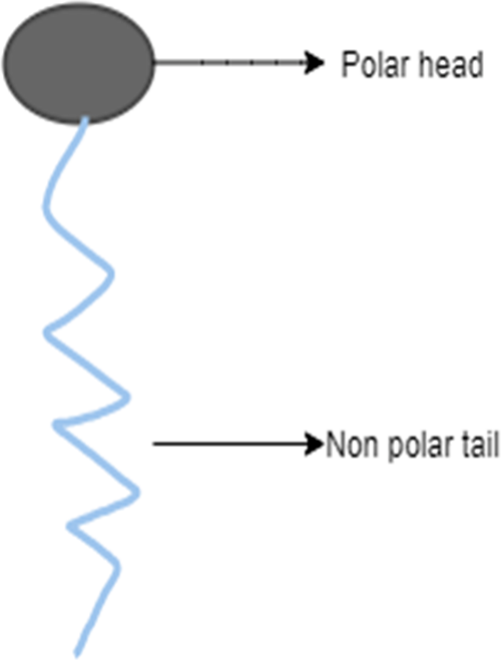
Schematic presentation of surfactant.

Surfactant, also known as a surface-active agent, reduces the surface tension of a liquid when added to it.^61,62^ Surfactants are amphiphilic, meaning they have a dual affinity, which is described as a polar–apolar duality from a physico-chemical perspective. The polar head of a surfactant comprises heteroatoms such as O, S, P, or N, which are found in functional groups such as alcohol, thiol, ether, ester, acid, sulfate, sulfonate, phosphate, amine, and amide.^63^ On the other hand, the nonpolar (apolar) group is a hydrocarbon alkyl or alkylbenzene-type chain, occasionally containing halogen atoms and even a few nonionized oxygen atoms. Surfactants can be obtained in both natural and synthetic forms. Oleo-chemicals are natural surfactants (vegetable or animal origin) that are obtained from sources such as palm oil or tallows. Petrochemicals are surfactants with a synthetic origin that are produced from petroleum.^64^ A surfactant’s typical structure is shown in Figure 5.

### 5.1 Types of Surfactants

Surfactants are mainly classified based on formal charges on the polar headgroup.

#### 5.1.1 Anionic Surfactant

An anionic surfactant is one in which a negative ion is present in the headgroup. Alkyl sulfates, alkyl ethoxylate sulfates, and soaps are the most widely used anionic surfactants. Carboxylates, sulfonates, sulfates, and phosphates are examples of anionic surfactants. These are utilized as hand-dishwashing solutions, all types of liquid laundry detergent, cleaning chemicals, and mobile phases in thin layer chromatography. They are especially suitable for highly concentrated products and industrial cleaning agents.^61^

#### 5.1.2 Cationic Surfactant

Cationic surfactants have a positive charge in the headgroup, such as alkyl trimethylammonium salt, alkyl ammonium salt, and alkylpyridinium salt. Cationic surfactants are frequently utilized in cosmetics as antifungal, antibacterial, anticancer,^65^ and antiseptic agents.^61^ It can alter the soil’s surface.

#### 5.1.3 Nonionic Surfactant

The absolute charges are not present in the nonionic surfactant. Due to the lack of an electrical charge, these surfactants are resistant to water hardness deactivation. Nonionic surfactants offer a wide range of applications in textiles, and they are widely utilized.^66,67^ The polyoxymethylene alkylphenols and polyoxyethylenates are the most prevalent kinds of nonionic surfactants. Detergents, solubilizers, and emulsifiers can all benefit from nonionic surfactants.^68^

#### 5.1.4 Zwitterionic Surfactant

**Figure 6:**
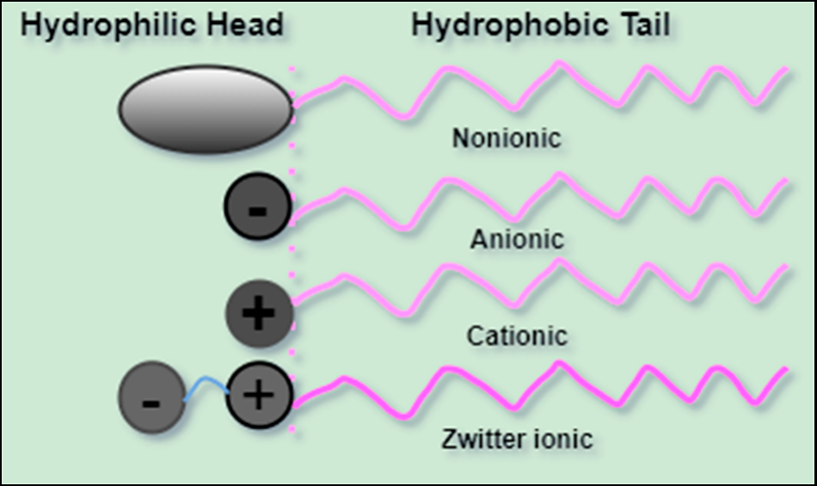
Schematic presentation of different types of surfactants.

Zwitterionic surfactant is a surfactant that has both positive and negative groups in the head portion (Figure 6). Alkyl betaine is a typical example. Because these surfactants are so mild, they are ideal for use in personal care and home cleaning products. Depending on the acidity or pH of the water, they can be anionic (negative group), cationic (positive group), or nonionic (no charge) in solution. Two charged groups of different signs may be present in these surfactants. While the positive charge is usually typically ammonium and the negative charge can come from a variety of sources (carboxylate, sulfate, sulfonate).^69^

### 5.2 Surfactant Aggregation

Micellization is caused by a precise balance of repulsive and attractive forces or by noncovalent interactions such as van der Waals force of attraction, hydrogen bonding interactions, or hydrophilic and hydrophobic interactions. The electrostatic repulsions between the headgroups are the primary source of repulsive forces in ionic surfactants. Even after decades of investigation, the actual nature of attractive interactions is unknown. For a long time, the fundamental attraction for micelle formation was considered to be the release of structured water from hydrophobic hydration layers surrounding the alkyl chain of the surfactant molecule (hydrophobic interactions). The values of the enthalpy (*H*_mic_) and entropy (*S*_mic_) of micellization at specific temperatures were used to support the significance of one of the two interactions. Positive *H*_mic_ and *S*_mic_ values were supposed to indicate the significance of hydrophobic contacts, whereas especially low *H*_mic_ values were thought to indicate that London dispersion interactions are the primary attractor for micellization. The reversible effort done by the solvent to rearrange and solvate the solute is denoted by G. Micelle production, on the other hand, is a complex process in which surfactant alkyl chains, surfactant headgroups, counterions, and water all play a crucial role. When London dispersion interactions are believed to constitute the dominant attractive factor for micelle formation, these results may be explained. The hydrophobic contact, which is mediated by water, appears to produce a clustering of hydrophobic units.^70,71^

### 5.3 Types of Surfactant Assemblies

The three most frequent surfactant configurations are, first, aligning on the solvent’s surface (causing a decrease in surface tension), second, micelles (formation spherical aggregates), and, third, a double-layer micelle, such as a vesicle. Surfactant assemblies such as micelles, microemulsions, hemimicelles, bilayers, and vesicles are all conceivable.^72^

#### 5.3.1 Micelle

**Figure 7:**
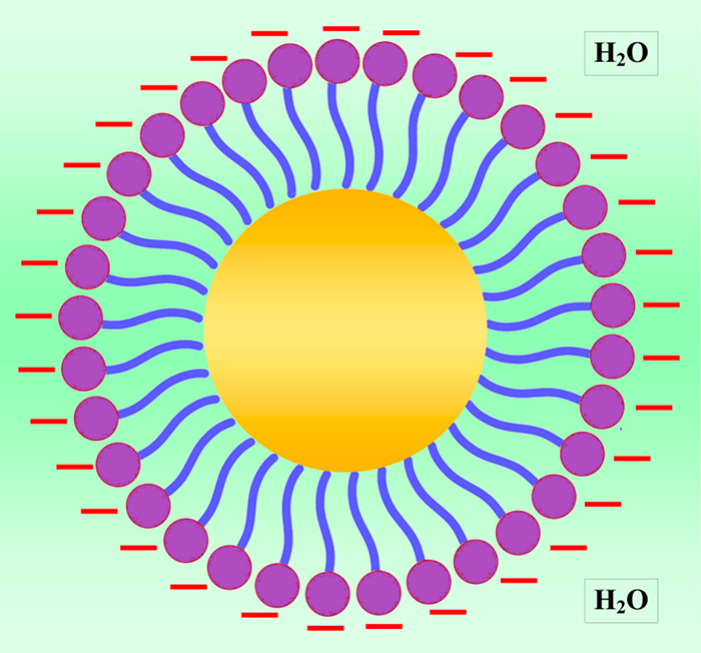
Schematic presentation of a micelle.

A micelle is a surfactant monomer reservoir. McBain used the term micelle in 1913 to characterize molecular aggregation in aqueous soap solutions. The surfactant molecules in a micelle arrange themselves such that the hydrophobic sections are away from water interaction and the hydrophilic parts are in contact with it (Figure 7). Depending on the size and structure of the surfactant, the rate of exchange of a surfactant molecule among micelle and bulk solutions might vary by many magnitudes.^73^

#### 5.3.2 Reverse Micelle

Reverse micelles are formed by the reversible production of association colloids from surfactants in nonpolar liquids. The polar groups of the surfactants are concentrated in the center of an inverted micelle and lipophilic groups, on the other hand, stretch toward and into the nonpolar solvent.^74^

#### 5.3.3 Monolayer

A monolayer is an organized layer of amphiphilic molecules with a particular, reversible affinity for a substrate at one end of the molecule, the “headgroup”. It also has a tail at the end of which is a functional group. These are made via chemisorption of hydrophilic “headgroups” from the vapor or liquid phase onto a substrate, followed by a gradual two-dimensional organization of hydrophobic “tail groups”.^75^

#### 5.3.4 Bilayer

**Figure 8:**
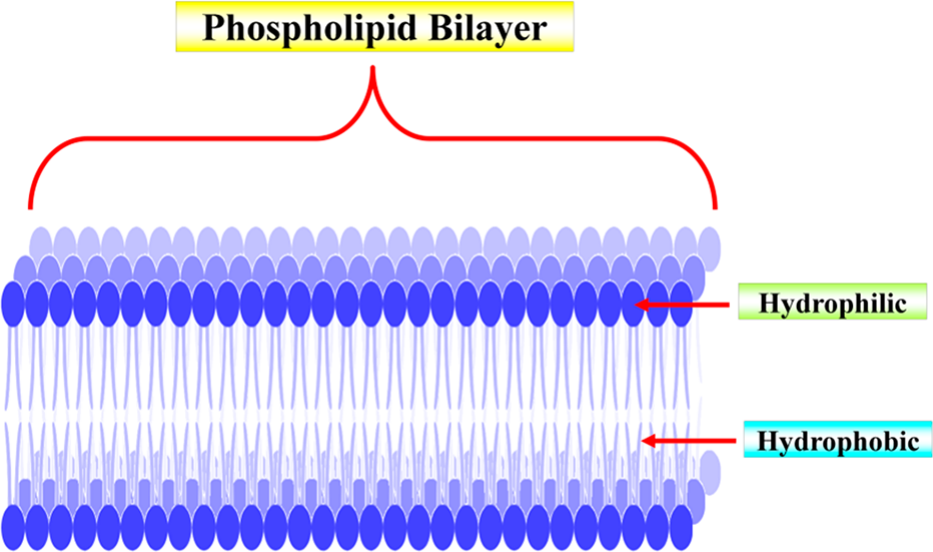
Schematic presentation of the bilayer.

A plate-shaped micelle is a membrane made up of two molecular layers with a diameter of 4–5 nm (Figure 8), and the micellar phase is liquid crystals.^76^ The spherical cage’s usual size spans from 200 to 1500 nm.

### 5.4 Characteristics of Micelles

#### 5.4.1 Critical Micelle Concentration

The critical micelle concentration (CMC) of a surfactant is the concentration range beyond which physical parameters such as electrical conductivity, osmotic pressure, surface tension, density, light scattering, refractive index, or the medium’s polarity abruptly change.^77,78^ The CMC is a key micellar quantity for studying amphiphile self-aggregation in solutions. CMC is the concentration at which the micelle begins to form, and it happens throughout a wide concentration range rather than a single point. The size of this range varies depending on the physical qualities that are being measured. The CMC, according to Corrin,^79^ is the total surfactant concentration at which a limited and constant number of surfactant molecules are in a structured format. According to Williams,^80^ the CMC is the surfactant concentration at which the micelle concentration would become zero if it continued to change at the very same rate as it does at a slight line of solution properties above and below CMC, but the accuracy of the measurement is dependent on the width of the concentration range that shows the change in physical properties. When the change is small, obtaining a unique point is nearly difficult. The CMC, according to Phillips,^81,82^ is the concentration that corresponds to the greatest change in the gradient of the solution characteristics vs the concentration curve.

#### 5.4.2 Factors Affecting Critical Micelle Concentration

The value of CMC is determined by a variety of factors. The following are some of the most significant factors.^83^

##### 5.4.2.1 Chain Length of Surfactant

The monomer has a stronger tendency to solubilize the more surface-active amphiphile. The CMC of a monomeric surfactant decreases as the total carbon chain of the surfactant grows longer. The number of carbon atoms “*n*” in anionic amphiphile unbranched hydrocarbon chains has a linear relationship with the CMC (eq 1)
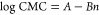
1where *A* and *B* are constants.

This association no longer holds for chains longer than 16 carbon atoms, and increasing the chain length has no discernible influence on CMC findings, probably due to the coiling of the chain. There is a strong link between the CMC of water and the POEO system in terms of extremely hydrophilic and hydrophobic portions.

##### 5.4.2.2 Additional Polar Groups

Double bonds (C=C) and chain branching tend to increase the CMC. It has been observed that the substitution of the CF_3_ group for the CH_3_ group of the surfactant hydrocarbon chain roughly doubles the CMC.^77^

##### 5.4.2.3 Headgroup of Surfactants

Changes in the hydrophilic portion of amphiphiles have a substantial impact on CMC, in general. The chain length of the surfactants has a much greater impact on CMC than the ionic head.

##### 5.4.2.4 Counterion

CMC decreases when the counterion is changed to one with higher polarizability or valence in traditional ionic surfactants.

##### 5.4.2.5 Temperature

Temperature has a far more complicated influence on the CMC of charged micelles. At lower temperatures, there is a reduction in CMC as the temperature increases. As the temperature increases, the CMC increases, as well. Thermodynamic data for micellization may be obtained by measuring changes in CMC as a function of temperature and pressure.

##### 5.4.2.6 Pressure

Proton NMR chemical shifts were used to determine the CMC of the nonionic surfactant C8E5 in deuterated water at 30 °C and pressures up to 350 MPa. The CMC has been discovered to grow with pressure up to around 150 MPa before decreasing at higher pressures.

##### 5.4.2.7 Hydrophilic Lipophilic Balance Number

The ratio of hydrophilic and hydrophobic groups is measured using the hydrophile–lipophile balance (HLB) number. Other approaches have been suggested by Griffin in 1949 and 1954 that the hydrophilic–lipophilic balance of a surfactant is a measure of its degree of hydrophilicity or lipophilicity and is determined by computing values for different areas of the molecule. It is supplied by nonionic surfactants.
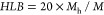
2*M*_h_ represents the molecular mass of the hydrophilic portion of the molecule, and *M* represents the molar mass of the whole molecule, yielding a value on a 0–20 scale. A molecule with an HLB value of 0 is fully hydrophobic, whereas a molecule with an HLB value of 20 is entirely made up of hydrophilic components.

#### 5.4.3 Additives

The additives are different. It may be an electrolyte, organic material, or a different surfactant.

##### 5.4.3.1 Electrolyte

The addition of electrolytes decreases the CMC. The CMC can be correlated with electrolyte concentration by an empirical relationship, according to a proposed equation (eq 3).
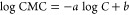
3where *a* and *b* are constants for a particular ionic group and *C* denotes the total concentration of the electrolyte.

However, the additive which breaks the water structure increases CMC.

##### 5.4.3.2 Organic Additives

There are two types of organic compounds that have a significant impact on the CMC of surfactants in aqueous solutions.

Class I: materials changing the CMC being entrapped into the micelle.

Class II: materials changing the CMC by modifying solvent–micelle interactions.

## 6 Surfactants Used in Different Types of Slurry Stabilization

The prime factor in the destabilization of a slurry is the interaction of solid particles among themselves rather than with the surroundings. The fundamental requirement of the slurry stabilization through dispersant (surfactant) development is, therefore, to promote a solid–water interaction over a solid–solid interaction. Solid particles such as coal,^27^ fly ash,^84,85^ or clay have both hydrophobic and hydrophilic regions. The surface charge of the naked solid particles, surface chemical alterations resulting from the adsorption of surfactant, and the orientation/organization of surfactant at the solid–water interface usually decide the nature of the solid surface. Surfactants have been widely used in the stabilization of different solid particles such as coal, fly ash slurry, clay, food materials, and liquid substances such as crude oil in slurry form and are reported in Table 3.

**Table 3:** Surfactants Used in Different Types of Slurry Stabilizations

name of the surfactant	type of slurry stabilization	reference
sodium polyacrylate and sodium styrene–sulfonate	coal–water slurry	Kakui and Kamiya^86^
SDS and CTAB	coal–water slurry	Gürses et al.^87^
sulfonated acetone formaldehyde resin and naphthalenesulfonate formaldehyde	coal–water slurry	Qiu et al.^88^
*Sapindus laurifolia*	coal–water slurry	Das et al.^26^
polyisoprene sulfonic acid soda (Dynaflow-K)	coal–water slurry	Dincer et al.^89^
*Acacia concina*	coal–water slurry	Das et al.^90^
grafted sulfonated alkali lignin polymers	coal–water slurry	Qin et al.^91^
acacia auriculiformis and carboxyl methyl cellulose	coal–water slurry	Das et al.^92^
ethoxylated nonylphenol surfactants and medium chain alcohols	crude oil-in-water emulsion	Santos et al.^93^
ethylene-vinyl acetate copolymer	crude oil-in-water emulsion	Taraneh et al.^94^
SMATWEEN	crude oil-in-water emulsion	Orazbekuly et al.^95^
Triton X-100	crude oil-in-water emulsion	Ashrafizadeh and Kamran^96^
sodium carbonate (Na_2_CO_3_)	crude oil-in-water emulsion	Ashrafizadeh et al.^97^
*Sapindus laurifolia* and SDS	fly ash water slurry	Behera et al.^34^
*Acacia auriculiformis* and sodium silicate	fly ash water slurry	Behera et al.^28^
CTAB	fly ash water slurry	Naik et al.^33^
*Acacia concinna*	fly ash water slurry	Pattanaik et al.^20^
sodium silicate	fly ash water slurry	Senapati et al.^98^
S40	limestone–water slurry	He et al.^99^
cocamidopropyl hydroxysultaine	limestone–water slurry	Alvarez et al.^100^
bis(2-ethylhexyl)sulfosuccinate sodium	clay–water slurry	Suzzoni et al.^101^
sucrose capric acid ester and sucrose lauric acid ester	food–water slurry	Krawczyk^102^

## 7 Dispersion and Rheology Study of IO Particle in the Presence of Surfactant

To obtain the appropriate slurry, it is important to use an effective dispersant in an appropriate quantity. The adsorption pattern of a dispersant is important because it affects the rheological behavior of a slurry.^103^ Surfactant’s vital role can be summarized as follows:

- Bring significant changes to the surface of the solid particles.
- Scheming the relative hydrophilic/hydrophobic properties of iron.
- Forming a three-dimensional structure that resists coagulation of slurry.
- This should be nonfoaming, water-soluble, and effective at low doses.
- It will have to work in conjunction with the stabilizer.

There’s no single factor that could fully explain the highly complicated rheology of mineral suspension. The rheology of slurry is significantly influenced by the physical and chemical characteristics of the slurry, such as the density, PSD, the morphology of particles, pH value, charge density, and slurry temperature.^104−106^ Dispersants are generally employed to control the rheology of slurry.^107,108^ By altering the surface properties of particles, a good dispersant could make interparticle forces completely repulsive. Surfactants in a solution lower the solution’s surface tension and/or interfacial tension. As a result, the surface tension of the solvent constantly drops as surfactant concentration gradually increases. At CMC surface activity of surfactant is maximum.^50^ This concentration generally decides the rheology of high concentration slurry because at or above this concentration viscosity reduction is maximum. Therefore, by measuring the CMC of surfactant, slurry transportation can be optimized. The research suggests that the repulsive force exerted on a particle in solution may be directly correlated with the magnitude of the zeta-potential.^109^ Many researchers have reported that the absolute value of zeta-potential larger than 30 mV was sufficient to maintain the particle. It is important to consider PSD while preparing a concentrated slurry.^110^ Flow challenges in the pipeline are caused by slurry with extremely fine particulate. Its dispersion is essential for creating a low-viscosity, highly concentrated, and stable CWS.^30^ Interstices or spaces between particles should be eliminated to achieve the maximum solid concentration with a viscosity that is within an acceptable range.^111^ So proper choice of a mixture of coarse and fine particles is needed for maximum solid concentration and economic pipeline transportation.

For the generation of ultrafine powders in manufacturing sectors, wet ultrafine grinding is becoming more and more attractive. Slurry rheology is considered to have a significant impact on the ability of industrial minerals to grind in wet ultrafine processing. He et al.^105^ investigated the rheology of IWS using a variety of dispersants (cationic, anionic, and nonionic) for slurry viscosity control. They postulated that poly(acrylic acid) or its salts with a molecular weight in the range of 5000 to 20,000 are the most commonly employed dispersants in slurry rheology. They postulated that for chemicals to act as dispersants, it is necessary to satisfy some required conditions, including the following. (a) The dispersants should adhere to the solid surfaces sufficiently to affect the slurry’s viscosity, and (b) the slurry’s viscosity has to be sufficiently high for the use of the dispersant to noticeably lower or reduce the slurry’s viscosity. (c) The dispersants must be nontoxic and biodegradable. (d) They must be consistent in their ability to reduce viscosity as a function of changing dispersant concentrations, pH values, water quality, and amounts of the shear present. (e) They should not adversely affect flotation, thickening, and pelletization or contaminate the resulting products and must be financially viable.

Magnetite and ferrosilicon particles are the predominant dense media particles involved in creating dense medium suspensions. While ferrosilicon is a synthetic iron and silicon alloy, magnetite is found as a natural iron oxide mineral. The stability and viscosity of thick medium suspensions are two of the most important factors.^112^ To reduce the viscosity of ferrosilicon and magnetite dense medium dispersion, polymeric dispersant (DP001) was used at various concentrations by Mabuza et al.^113^ A tiny amount of polymer was found to reduce the viscosity of the medium by about 20%. Gravities and slime concentrations are present in the slurry, allowing for reductions of up to 50%. At all shear rates examined, the inclusion of surfactant DP001 in dense media suspensions containing magnetite reduces the viscosity of the dispersions by a significant amount, but the impact of slimes on raising the viscosity of a ferrosilicon dispersion can be reduced by up to 50% by applying the same reagent. When compared to additions at lower concentrations, large amounts of DP001 appear to have minimal influence on viscosities, with the optimum load appearing to be approximately 1 g/kg of solids. Although the majority of the initial studies with DP001 were done at a lower density, the results are still important.

Four different types of dispersant (sodium hexametaphosphate, quick lime, hydrated lime, and acti-gel) in the concentration range from 0.05 to 2% were used for dispersion and rheological behaviors of IWS at varying iron concentrations of 18.8, 22.1, and 25.8%.^54^ It has been investigated that minimum shear stress and fluidity were achieved with a 2% additive dosage of quick lime at 18.8% IO concentration. On the contrary, addition of hydrated lime increases the shear stress, viscosity, and flow behavior index, but the addition of sodium hexametaphosphate (1.5%) minimum viscosity and shear stress obtained the solid concentration of 18.8%. With the increase in the amount of sodium hexametaphosphate (2%), more solid concentrations are achieved (22.1 and 25.8%) with the lowest viscosity and yield stress. When quick lime and hydrated lime were employed in all solid percentages and additive concentrations, the pH values of the slurries were noticeably increased. Acti-gel, however, has no impact on pH because of its inert nature.

**Figure 9:**
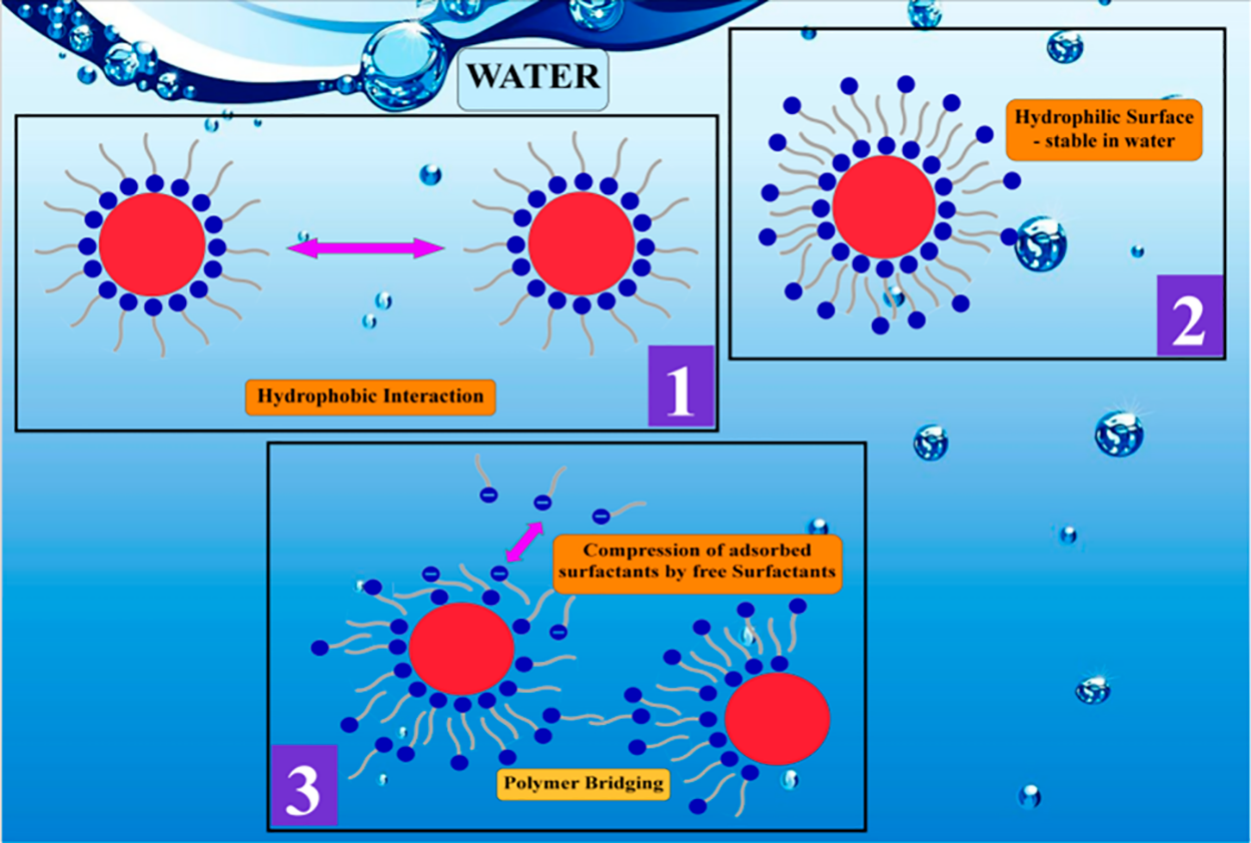
Adsorption of SDBS on the magnetite (Fe_3_O_4_) surface.

Sodium dodecyl benzenesulfonate (SDBS) an anionic surfactant was found to be an effective stabilizing agent for iron oxide particles. Lee et al.^25^ used surface tension, zeta-potential, and contact angle measurements to evaluate the interaction between magnetite particles and SDBS. It has been demonstrated that the pH of the medium and the surface charge of the magnetite particle both significantly affect adsorption. At pH values below the isoelectric point, a significant amount of adsorption occurs as a result of the electrostatic attraction force between the negatively charged magnetite particle and the positively charged anionic SDBS. Adsorption is restricted at pH levels over the isoelectric point by electrostatic repulsion forces caused by the fact that both magnetite and SDBS surfaces are negatively charged. The adsorbed layer is said to convert from a monolayer to a bilayer and then to a compressed layer onto agglomerated particles created by polymer bridging as the amount of SDBS increases (Figure 9). The median diameter of the SDBS-containing suspension drops with increasing pH at first, reaching its minimum value at pH 4, before rising again with increasing pH. The decrease in median diameter up to pH 4 is appropriately explained by the decrease in zeta-potential due to SDBS adsorption. The contact angle lowers slowly at first as adsorption increases, then drops dramatically, then bounces back to a big value with more adsorption. It can be separated into three sections, as shown in the diagram shown in Figure 9. An increase in the contact angle value shows the stabilization of iron oxide particles. Similar types of work have been carried out by Suzzoni et al.^101^ They investigated the interactions between kaolinite and the common anionic surfactant bis(2-ethylhexyl)sulfosuccinate sodium. First, it is realized that surfactant adsorption isotherms on kaolinite depend on pH. The structure of the adsorption isotherm in acidic media reveals that, first, a surfactant monolayer is adsorbed on the positively charged edge surfaces below the CMC, and, second, a bilayer is adsorbed on the edge surfaces above the CMC. This assumption is supported by hydrophobicity experiments, which show that the surface changes from hydrophilic to hydrophobic in the first scenario and vice versa.

Wang et al.^114^ investigated the surface adsorption behavior and mechanism of a mixed surfactant system, sodium oleic (SO), and SDBS on magnetite particles using infrared, thermogravimetric, and zeta-potential analyses. The adsorption isotherms model for SO on magnetite particles show excellent consistency with the Langmuir type, but the adsorption formulation for SDBS on magnetite particles coated with SO shows remarkable consistency with the Freundlich method. The detailed mechanism of adsorption can be explained as follows. As a result of the interaction between the magnetite particles and the SO carboxyl, a SO monolayer was initially formed on their surface by the chemical adsorption process. The alkyl chain of SO extended into the liquid, giving the particle’s surface a hydrophobic coating. Physical adsorption occurs when the SDBS molecules are absorbed onto the surface of magnetite particles coated with SO through van der Waals attraction with the polar end of SDBS molecules stretching into the water, and the particle’s surface became hydrophilic.

Because of its capacity to remediate a wide range of organic and inorganic pollutants, nanoscale zerovalent iron (NZVI) particles have lately sparked a lot of importance.^115^ However, the lack of stability of NZVI water suspensions makes field implementation of this technique difficult. This study shows that adding 6 g/L of xanthan gum bio dispersant to highly concentrated NZVI slurries (15 g/L) may stabilize them for more than 10 days. Modification of ionic strength was used to produce stability against aggregation and sedimentation, which is primarily related to the creation of a viscous gel with shear-thinning behavior.^115^ The most common method for reducing particle aggregation and producing stable NZVI dispersions is to increase repulsive interaction, which can be accomplished in three ways: enhancing the surface charge of nano iron (electrostatic stabilization), attempting to keep colloids at a safe distance (steric stabilization), or a combination of both.

The combined effect of particle size, blend ratio, and selected bioadditives on the rheological behavior of Indian IO samples in a slurry range of concentrations of 60–75% was examined by Senapati et al.^116^ In the presence of two biodispersants, Indian spinach (*Basella alba*) and Bellyache bush (*Jatropha gossypifolia* Linn), having dosages of 0.8–1% (w/w) of total solids and specific multimodal IO sample with a broad PSD, showed a significant reduction in slurry viscosity, yield stress, and improved solid packing compared to monomodal PSD. They further investigated that the creation of loosely packed flocculation and the immobilized water in the slurry occurs with an increase in iron concentration in the slurry, which increased particle–particle and particle–fluid friction The lignans, saponins, and tannins found in these two bioadditives are thought to modify the iron surface for excellent stabilization of slurry. Bellyache bush extracts were shown to be more beneficial than Indian spinach at reducing IO slurry yield stress and fluidity. The findings show that combining fines with coarse IO at a regulated particle size distribution, together with tiny doses of low-cost additives, can be used to prepare and transfer high-concentration IO slurry with enhanced results.

Wang et al.^117^ demonstrated an effective technique to obtain innovative NZVI surface modifications by employing Pluronic F-127. Pluronic F-127 is soluble in water and amphiphilic in nature. It is a copolymer of poly(ethylene oxide)–poly(propylene oxide)–poly(ethylene oxide) (PEO–PPO–PEO) and is biodegradable. Due to the molecular architecture of Pluronic F-127, which comprises two highly hydrophilic PEO groups joined by a hydrophobic PPO unit in the center, Pluronic F-127 micelles are easily formed in the liquid phase. The unique chemical structure of Pluronic F-127 makes it suitable for being used to modify NZVI. The scanning electron microscope, transmission electron microscope, and X-ray powder diffraction (XRD) images shows that the particle size of Pluronic F-127-modified NZVI was reduced compared to bare nano-zero-valent iron particles (BNZVI), while the dispersity and antioxidizability of NZVI were increased. Furthermore, the appearance of a C–O bond in the XPS and Fourier transform infrared patterns of NZVI revealed that Pluronic F-127 micelles have been successfully coated on nano-zero-valent iron. The PPO core in this study can contain a significant number of Fe nanoparticles, while the PEO shell makes sure that the micelles stay dispersed. As a result, the surface coating of Pluronic F-127 greatly increases the dispersity and reactivity of NZVI.

A novel method of stabilizing iron particles by guar gum surfactant by Gastone et al.^57^ Guar gum is a food-grade, environmentally benign natural polysaccharide that is widely used as a thickening ingredient in a variety of culinary, medicinal, and industrial products. Guar gum solutions are shear-thinning non-Newtonian fluids with high viscosity in static circumstances and low viscosity in varying loads. The high zero shear viscosity, in particular, ensures microscale zero-valent (MZVI) dispersion stability, lowering particle sedimentation rates and allowing storage and field operations. When NZVI and MZVI are distributed in pure water, they are both unstable. Because of strong magnetic particle–particle interactions,^118^ agglomeration and subsequent sedimentation of NZVI particles can be successfully avoided by altering the surface characteristics of the particles with polymeric coatings^119^ or partial coatings with other metals.^120^ On the other hand, MZVI has a higher colloidal instability due to its greater size that causes rapid sedimentation. As a result, rather than changing the surface characteristics of the particles, a change of the dispersion fluid is required.

By observing magnetite nanoparticle settling behavior and analyzing transmittance, zeta-potential, and particle size, the influence of polymeric dispersants on the dispersion stability of MNPs in a water solution was determined.^121^ The dispersion stability of MNPs did not increase linearly in proportion to dispersant concentration, and the crucial concentration of dispersant for improving MNP dispersion ability is in the region of the concentration ratio of dispersant to MNPs in the range 0.1 to 0.01.^121^ The zeta-potential of MNPs decreased as polymeric dispersant concentrations increased, resulting in the production of aggregated MNPs at about 0.01. This difference suggests that the interaction between adsorbed polymers and other particles counteracts each other.

In comparison to bare iron oxide particles in an aqueous solution, the charged Fe_2_O_3_ particles were effectively dispersed by oppositely charged stabilizers via electrostatic contact and exhibited good long-term stability. When Fe_2_O_3_ particles were coated with hydrophobic surfactants such as SDS and poly(allylamine hydrochloride), the PSD was broad, but particles coated with polyacrylic acid and poly-4-vinylbenzenesulfonate sodium salt have a packed PSD. The interaction between the hydrophobic surfaces of metal oxides and the hydrophobic long alkyl chains of the surfactant caused the charged colloid and surfactant suspensions to flocculate.^122,123^

According to Jones and Horsley,^124^ chemical additives increase the flowability of slurries, allowing them to be pumped at much higher solid concentrations and thereby lowering water utilization. As a result, one of the most important considerations in the preparation of slurries for shipping should be the selection of appropriate additives. According to Jones and Horsley’s concept, sodium tripolyphosphate attaches to iron oxide particles, and because the polyphosphate is a multicharged anion, it changes the surface charge of iron oxide from positive to negative. As a result, the shear-thinning suspension exhibits almost no yield stress, behaving like a Newtonian fluid. Electrostatic repulsion happens as a result of the iron particle accumulating too much negative charge. According to Schick and Villa,^125^ chemical additives must be selected to achieve appropriate viscosity, zeta-potential, and desirable stability. To modify the rheological characteristics of IO, a variety of chemical agents have been employed in the mining sector at various stages of processing. These chemicals could have worked as dispersants, flocculants, surfactants, or antisettling agents, among other things.

Focusing on molecular design, surface chemistry, polymer chemistry, and a binding system failure model, Qui et al.^10^ explored the functionalities and molecular design of suitable organic binders for palletization and dispersion of IO. Their research revealed that the −COO and −OH groups of organic binders are excellent polar or hydrophilic functional groups, respectively. They concluded that suitable organic binders for pelletizing IO should have structurally adequate polar or hydrophilic functional groups.

Marcos and Antonio^126^ investigated how the slurry’s rheological behavior, as well as the agglomeration and dispersion conditions, influenced wet nanoscale milling of IO concentrates. They investigated that adding lime to the fluid increased specific energy consumption, as well as a considerable rise in yield stress and fluid consistency index. They also further postulated that the mechanism of dispersion is based on increasing the amount of negative charge on the particle surface which keeps the electrical double layer sufficient apart from each other.

Comminution stages are problematic in the mining sector because of their low mechanical accuracy and greater energy demand, resulting in greater operating costs in concentrators. Viscosity, not solids percentage, governs the relationship between the pulp and the milling medium inside the mill. According to Vieira et al.,^127^ the dispersion degree was estimated using dispersing agent NaOH, a sedimentation tube, flux curve analysis, and the coefficients of the Herschel–Bulkley and Bingham models. According to them, adding 300 g/t of lime and raising the pulp pH from 7.3 (natural pH) to 10.0 enhanced the rheological behavior of IO pulps. As a consequence, the particle dispersion percentage in the pulp increased from 3 to 28%, while yield stress, apparent viscosity, and cost of energy dropped by 17.4%. The grinding ability, fine grinding efficiency, and energy demand of industrial minerals in the wet grinding process were all influenced by the rheology of the slurry. They also investigated that calcium ion has no impact on the specific energy consumption and Blaine surface area generation because no differences were found by experimenting using processed water or distilled water. Therefore, calcium ions are not significant enough to have an impact on the regrinding performance.

**Figure 10:**
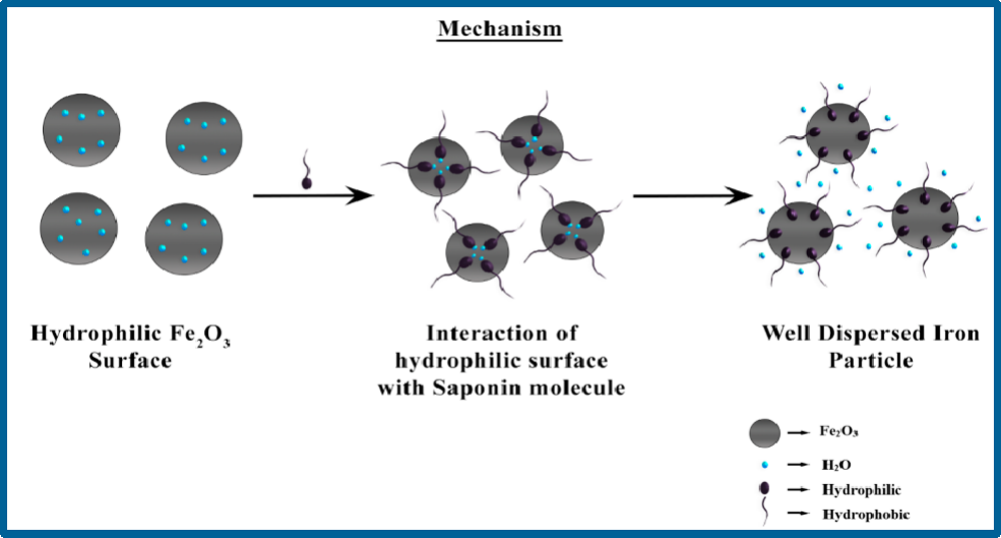
Mechanism of transportation of IWS. Reprinted with permission from ref (11). Copyright 2022 Elsevier B.V.

The rheology of the slurry is determined by the kind of solid particles, molecular weight, molecular structure, side-chain length, hydrophobic to hydrophilic group ratio, composition, and amount of the polymer.^128^ The concentration of ionized functional groups (carboxylic and sulfonic) that project to the outer side of the water medium increases as the molecular weight of the dispersant increases thus increasing the strength of the electrical double-layer repulsion. This increased repulsion between the particles. Thus, increasing the molecular weight of the surfactant, the viscosity of the slurry decreases. The surfactant’s alkyl groups adsorb onto the coal’s hydrophobic site, producing a partial negative surface charge that attracts counter cations to the interface, forming an electrical double layer.^129^ When such an electrical double layer approaches each other, it generates steric repulsion, preventing coal particle aggregation. To stabilize the slurry, a nonionic surfactant can also be utilized as a dispersing agent. In this case, the mechanism of stabilization is quite different from that of an anionic surfactant.^129^ The following explanations are given for the stabilization of slurry by a nonionic surfactant. There are two ways for the surfactant to adsorb to the particle surface. In the first probable method, the surfactant’s hydrophilic site may adsorb on the coal surface’s hydrophilic site, directing the surfactant molecules’ hydrophobic sites toward the aqueous phase of slurry. The hydrophobic sites of the surfactant may adsorb on the hydrophobic particle surface in the second possible process, directing the hydrophilic site toward the aqueous phase. Because of hydrogen bonding between the polyethoxylene chain and the water molecule, the quantity of water at the particle’s surface increases, thereby lowering the viscosity. Saponin a nonionic surfactant extracted from the fruits of *Sapindus laurifolia* (*S. laurifolia*) as a stabilizing and dispersing reagent in the storage and transport of IO particles was studied by Behari et al.^11^ IWS rheological behavior has been investigated concerning saponin doses, IO concentration, pH, temperature, and the shear rate-shear stress relationship. As the saponin concentration increased, the slurry’s viscosity and yield stress value dropped. The maximum reduction in viscosity (1200 to 398 mPa·s) occurred at saponin concentrations of 0.021 g/cc (Aq. Extraction process) and 0.011 g/cc (Chem. Extraction process), which are just above the CMC (0.017 g/cc for aqueous and 0.008 for chemical extraction process) of saponin. The apparent viscosity and yield stress of the IWS were at their maximum at the isoelectric point because there was no movement of IO particles at this point. The increase in viscosity can be correlated to IWS’s isoelectric point, which was about pH 6.8. Behari et al. developed a mechanism (Figure 10) for analyzing the interaction of *S. laurifolia* and IO particles. Because of the presence of the hydrocarbon chain, when the surfactant molecule adheres to the IO surface, the water molecules are expected to be desorbed from the mineral surface. This procedure is carried out until the CMC is reached. As a result, a well-dispersed IWS is produced, which prevents IO–IO interaction. The economic effects of the dispersant on the transport cost of the IWS pipeline are estimated based on slurry head loss, solids conveying rate, hydraulic power need, and specific power consumption. The addition of *S. laurifolia* to IWS reduced head loss, hydraulic power, and specific power consumption substantially.

Sun et al.^130^ investigated the effects of iron concentration, PSD, temperature, and the dispersant dosages (sodium hexametaphosphate) on the flow behavior and the bio-oxidation process of pyrite slurry. They explored the possibility that, with low solid loading, the comparatively large spacing between mineral particles may result in negligible interparticle forces. As the solid concentration increased, interparticle interactions became more prominent, and slurry viscosity increased. They also correlated the dispersant concentration with the stability of slurry. The fluid is acceptable up to 0.05 wt % of sodium hexametaphosphate, but when the concentration is increased to 0.1 wt %, there is a noticeable rise in viscosity. The DLVO hypothesis can be used to explain this, which states that as the dispersant dose grew, electrostatic stabilization caused the saturation adsorption on the pyrite surface. The viscosity of the slurry increased as a result of the excess dispersants still present in the slurry which could compress the electrical double layers.^109^ The electrostatic repulsion and DLVO forces between the pyrite particles were increased by the addition of 0.05 wt % of sodium hexametaphosphate. This is further confirmed as the absolute value of the pyrite zeta-potential increased from 20.7 to approximately 36 mV.

The relation between alkalinity and viscosity with solid concentrations ranging from 20 to 50% (by weight) was studied by Singh et al.^131^ At shear rates ranging from 0 to 600 s^–1^, slurry exhibits non-Newtonian behavior up to 30% solid concentration. The increase in alkalinity of the slurry with increases in solid concentration may be due to the reducing nature of iron. This is because iron present in the +2-oxidation state may be oxidized to the +3 state and enhance the reduction of oxygen to OH^–^ ions in the suspension. Thus, the creation of a sufficient number of OH^–^ ions in the slurry increases the zeta-potential and reduces the apparent viscosity.

It has been investigated by Sahoo et al.^132^ that microwaving of IO improves the rheological properties of IWS significantly. They investigated rheological studies for Indian IO conducted in a microwave oven at a power level of 900 W and with exposure times of 30, 60, 90, and 120 s. When compared to untreated ore, microwave-treated ore has improved rheological characteristics which may be because treated ore has a lower density than untreated ore.

An empirical equation was developed by Meikap et al.^133^ to predict the slurry viscosity. It has been investigated that apparent viscosity grows exponentially with the volume concentration and solid particle size. The resultant empirical equation for apparent viscosity (*Y*) is defined in terms of coded components in eq 4.
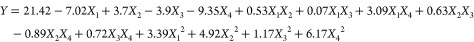
4Particle diameters (*X*_1_), solid contents (*X*_2_), microwave duration of exposure (*X*_3_), and shear rate (*Y*) as a function of apparent viscosity (*X*_4_). The synergistic impact is indicated by a positive sign in front of the term, and the antagonistic effect is indicated by a negative sign.

The fluid flow of the heterogeneous slurry in the pipeline was studied by Mukhtar et al.^134^ Their research aimed to see how concentration and velocity affected the relative pressure drop of slurry suspension of IO (sp. gravity 4.2) and zinc tailing (sp. gravity 2.6). Experiments were conducted out on a horizontal 90° pipe bend with a radius ratio of 4 and a radius of curvature of 21 cm at speeds ranging from 1 to 3 m/s and concentrations of IO ranging from 10 to 40% (by weight) for IWS and 30–45% (by weight) for zinc tailing slurry, respectively. IWS indicated Newtonian behavior up to 30% concentration, whereas zinc tailing slurry showed Newtonian behavior up to 40% concentration, according to rheological results. The pressure drop value is more for both zinc and iron slurry at lower speeds and appears to remain consistent as speed increases.

Studies were carried out to investigate the rheological properties of IWS by blending fine particles with coarser particles.^135^ By combining finer particles with coarse (53–75 μm) a bimodal slurry suspension of IO was created which shows pseudoplastic behavior, similar to that of unimodal suspension but with improved rheological behavior. It was discovered that when the fraction of smaller particles in slurry grows from 10% to 30%, the perceived viscosity of the slurry reduces and the blending of 30% finer particles resulted in the highest reduction in viscosity. The reduction in apparent viscosity is due to the lowering of surface tension and interparticulate forces.^136,137^

The current study aims to generate an extensive experimental data set from the pilot plant test and CFD analysis for a better understanding of the flow behavior of IWS pipelines flow.^138^ The report presents experimental data from 12 m long iron ore slurry flow through a 105 mm diameter pipe with flow rates ranging from 1.35 to 5.11 m/s and efflux concentrations ranging from 2.63 to 31%. The acquired findings are validated using a CFD model that is appropriate for the situation. In addition to utilizing simulated findings, a qualitative study of iron ore slurry flow instances has been reported.

Kaushal et al.^12^ carried out the computational fluid dynamics to investigate the flow behavior of a high concentration IWS flowing through a pipeline. The tests were conducted on a 3 m long horizontal pipe with a diameter of 54.9 mm. Glass beads with a mean particle diameter of 125 μm and a flow velocity of up to 5 m/s were used in the experiment. The efflux concentration varied from 1 to 50% (by volume) in the presence of sodium hexametaphosphate as a dispersant. Applying two models, namely, Eulerian and mixture models, Kaushal et al.^13^ estimated the pressure drop and velocity distribution at various iron and surfactant concentrations.^13,139^ Sodium hexametaphosphate being an anionic surfactant developed an intense negative charge on each iron particle, which caused particle–particle repulsion in IWS. Therefore, apparent viscosity, pressure drop, and yield stress were reduced drastically.

## 8 Conclusion

The use of minerals poses challenges that are more significantly addressed by the enhanced processing techniques of mineral sources and their prospective produced technologies. IO fines play an important role in sintering and palletization. The easy transportation of IWS from the mine to the steel and pellet factory, as well as its easy storage in the steel and pellet plant before use, makes this procedure more viable than the traditional one. Currently, a large amount of IO fines has been transported from the mining site to the plant through the pipeline, which is both economically and environmentally beneficial in comparison to the conventional transport system. Slurry pipeline systems are widely utilized around the world as a feasible alternative to large-scale solids transport via pipelines for conveying minerals such as fly ash, IO, coal, lime stone, copper concentrate, zinc tailings, and other materials. Apart from, the ease of distribution via pipeline, another main benefit of IWS is the reduction of iron dust explosions and the pollution generated by them, which might not only minimize health risks but also dramatically lower the lifetime risk among employees engaged in iron processing operations. Surfactants can increase the stability of IO particles by inducing electrostatic or steric repulsion when adsorbed on them. Thus, a well-dispersed slurry is formed in comparison to bare IO particles. The surface-modified iron particles coated with charged surfactants showed good long-term stability, which is an important factor for long-distance pipeline transportation. PSD of IO significantly affects the economy of slurry transportation by increasing the packing efficiency and decreasing the viscosity of the slurry. In addition to the effect of surfactant, PSD, temperature, IO concentration, pH, etc. have an important role in improving the flow behavior of IWS. The selection of the equipment and the estimation of corrosion-erosion are equally important to the hydraulic design. Future works should be more focused on minimizing corrosion-erosion during long pipeline transportation of IO and the extraction and transportation of iron resources that are located far away. From a safe and economical perspective, more research should be carried out on stabilizing IWS in the presence of natural surfactants.

## Abbreviations

IWS: iron water slurry
CMC: critical micelle concentration
PSD: particle size distribution
SDBS: sodium dodecyl benzenesulfonate
DDAB: didodecyl ammonium bromide
SDS: sodium dodecyl sulfonate
IO: iron ore
TFC: transitional fines content
